# What factors influence the average length of stay among stroke patients in a Nigerian tertiary hospital?

**DOI:** 10.11604/pamj.2017.26.228.12249

**Published:** 2017-04-25

**Authors:** Olasheni Abdul-Afeez Somotun, Kayode Omoniyi Osungbade, Oluwaseun Oladapo Akinyemi, Taiwo Akinyode Obembe, Folashayo Ikenna Adeniji

**Affiliations:** 1 Association for Reproductive and Family Health, Uyo, Nigeria; 2 Department of Health Policy and Management, College of Medicine, University of Ibadan, Ibadan, Nigeria

**Keywords:** Length of stay, stroke, hypertension, health systems development

## Abstract

**Introduction:**

Increased emphasis is being laid on ensuring that health resources are efficiently utilized, especially in resource-constrained settings such as in Nigeria. One of the main indices of how efficiently a health institution is being run is Length of Stay (LOS), which is likely to be higher in chronic diseases such as stroke and diabetes. Stroke is a chronic disease that is currently on the rise in Low and Middle income countries (LMICs) who are also characterized by constraint of health resources. This study seeks to determine the LOS of stroke patients as well as factors that affect it.

**Methods:**

A retrospective analysis of health records of stroke victims admitted into the medical wards of the University College Hospital, Ibadan between January 2012 and December 2014 was conducted. Data on sociodemographic information, comorbidities and risk factors were extracted while LOS was calculated by counting the number of days the patient was admitted. Analysis was carried to using SPSS.

**Results:**

A total of 143 records were used in the final analysis with 53.1% of them being males and having a mean age of 61.5 ± 14.2 years. More than half (53.8%) of the cases were ischemic strokes. The average length of stay was 13.7 ± 8.9 days while bivariate analysis showed that a greater proportion of cases who consumed alcohol, had diabetes and hypertension had LOS of over 7 days than those who did not. However, these differences in proportions were not statistically significant (0.310<*p*<0.883).

**Conclusion:**

LOS of stroke patients in Nigeria was shown to be prolonged especially when compared to similar settings in West Africa. The high prevalence of some of the risk factors of stroke such as diabetes mellitus indicates that policy and advocacy to drive changes in lifestyle are necessary to reduce the incidence of stroke and its consequent burden on health systems.

## Introduction

The efficiency with which health institutions are run, especially in resource-constrained settings continue to play a key role in determining how accessible health services are to the members of the public [1]. The importance of achieving improved efficiency of resources has been emphasized by health policy makers in the African region. The outcome statement of the WHO member states of the African Region provides an example of the increased emphasis being laid upon efficiency in utilization of health resources [2]. Similarly, improved efficiency has been touted by the World Health Organization (WHO) as one of the four overarching goals of health systems [3, 4]. Length of stay (LOS) continues to remain one of the most popular indices used in assessing hospital performance and efficiency. Length of stay has been defined as the period from the day of admission to the day of discharge or death [5, 6]. Studies involving LOS are usually aimed at exposing areas of inefficiency in hospital performance for appropriate rectification [2]. Stroke is defined by the World Health Organization as a syndrome of rapidly developing clinical symptoms and signs of focal (or global) loss of cerebral function with symptoms lasting 24 hours or more with no apparent cause other than vascular [7, 8]. Stroke is a costly disease from human, family and societal perspective. In terms of the human costs, stroke is a leading cause of death and disability. Every year, about 16 million first ever strokes occur in the world, causing a total of 5.7 million deaths [9]. Particularly worrying for countries such as Nigeria is the fact that stroke, unlike in previous decades, is now on the rise in low and middle income countries [10, 11]. For example, while the incidence of stroke decreased in high income countries between 2000 and 2010, the incidence in LMICs increased by as much as 22% and for the first time, exceeded that of high income countries by more than 20% [8]. In addition, about 85% of all stroke deaths are registered in low and middle-income countries, which accounts for about 87% of total losses due to stroke in terms of disability-adjusted life years (DALYs) [7].

In addition to the human costs, stroke has also been documented to exert tasking demands on the health system resources available. In Israel, stroke patients have been found to be admitted into the hospital for an average of between 7 days and 14 days with longer LOS reported in older stroke patients and those with other complication [12]. This is longer than the average of 5.5 days reported across all diseases in European countries. Also, in the European Union, stroke is responsible for as high as 12.1 in-patient days, second only to mental and behavioral disorders, emphasizing its toll on the health system inputs [13]. Studies have however demonstrated that the severity of stroke in patients can vary significantly based on several other factors such as age, presence of co-morbidities as well as other lifestyle characteristics [2, 5, 11, 14]. Considering that these factors can significantly affect hospital LOS, which in turn can impact on performance and efficiency, it becomes necessary for policy makers and health planners to understand the relationship between these factors and LOS among stroke patients as this will assist them in allocation of resources on patients at risk and allow stroke care units to develop better interventions to reduce prolong hospital stay and help control medical costs. This study seeks to provide this information by assessing the factors that affect LOS among stroke patients in a Nigerian tertiary hospital.

## Methods

**Study setting**: Ibadan is the capital of Oyo State, Southwest Nigeria. It is the third largest metropolitan area, by population, in Nigeria after Lagos and Kano with a population of 1,338,659 according to the 2006 census. The city's total area is 1,190 sq. mi (3,080km^2^). The principal inhabitants of the city are the Yoruba, with clusters of Igbos and Hausas living in several areas. University College Hospital (UCH), the premier tertiary hospital in the country, is strategically located in Ibadan. The physical development of the hospital started in 1953, but was formally commissioned in 1957. The hospital has 850 bed spaces and 163 examination couches. The current bed occupancy ranges from 55-60%. Stroke patients are usually admitted through the accident and emergency units and medical outpatients' clinics and subsequently transferred to the medical wards within 24 hours. There are 158 bed spaces available in the medical wards from where the stroke patients were treated.

**Sampling procedure**: The case records of stroke patients used for this study were stored in the medical outpatients' health records department. A retrospective descriptive hospital study of stroke patients admitted to the medical ward of UCH Ibadan between January 2012 and December 2014 was conducted. The case records of patients with clinic diagnosis were retrieved and reviewed, having obtained permission from the Chairman Medical Advisory Committee (CMAC) of the hospital. These were case notes of patients admitted into the accident and emergency unit and medical wards. A total sampling of all the case notes with a diagnosis of stroke was done. A checklist was designed to extract relevant clinical data from the case records. The checklist recorded the age, sex, date of admission, stroke risk factors etc.

**Measures**: Hospital bed days were calculated using the admission and discharge dates by counting the days within the period of stay for each patient. Hypertension was recorded as blood pressure higher than 139/90mmHg. Diabetes mellitus was recorded when the patient's blood sugar was above the normal levels. Stroke was defined as a clinical syndrome of sudden onset of rapidly developing symptoms and signs of focal or global cerebral deficit with symptoms lasting more than 24 hours or leading to death with no apparent cause other than vascular origin. The stroke types were classified using the international classification of diseases 10th revision (ICD 10). The following are the codes of typical stroke diagnoses: Subarachnoid hemorrhage (ICD 10:160), intracerebral hemorrhage (ICD 10:161), ischemic stroke (ICD 10:163) and stroke unclassified (ICD 10:164). The educational status of the patients was not captured in the case records, but the occupational status was available. Also payment methods were not available at the time of research.

**Length of stay**: The length of stay of each stroke patient was calculated from the day of admission to the day of discharge. The average length of stay (ALOS) was calculated as the total number of inpatient bed days (H) divided by the total number of admissions (discharges and deaths). Average length of stay is calculated as: average Length of Stay (ALOS) = H/ (D+d) [15] H= Total number of bed days (inpatient) in the year (stroke) (D + d) = Number of discharges (D) and deaths (d) in the same year

**Inclusion and exclusion criteria**: Health records with complete information of all patients with a diagnosis of stroke patients that reported and were admitted to the hospital within the study period were eligible for inclusion in the study. Health records of admitted stroke patients with incomplete data vital for the study were excluded.

**Data analysis**: All data were inputted and analyzed using the Statistical Package for Social Sciences (SPSS) version 20.0. Frequency, means and standard deviations were generated using descriptive statistics. Chi-Square was used to compare categorical variables. Statistical significance was fixed at P< 0.05. Serial numbers were assigned to each checklist and also inputted into the data view of the SPSS file for easy manipulation of data.

**Ethical considerations**: Ethical clearance for the study was obtained from the UI/UCH Ethical Review Committee. An application for data collection was submitted to the Chairman Medical Advisory Committee (CMAC) of UCH, Ibadan. A signed acceptance letter from the CMAC was sent to the Heads of Departments. Data collected were kept strictly confidential. Data that could identify the patients were recoded using serial numbers formulated during collection.

## Results

There were a total of 143 records with the majority of them (76; 53.1%) being male. The mean age at presentation was 61.5 ± 14.2 years. The highest proportion of patients was found among those 70 years and older while just 8 respondents, representing 5.6% of the sample were not currently married. Over half of the cases (53.8%) were ischemic strokes making it the most common while hemorrhagic stroke was the least commonly (18.2%) diagnosed stroke Table 1. More than three-quarters (76.2%) of the patients had earlier presented with cases of hypertension while about one in ten (10.5%) of them had suffered earlier episodes of strokes. Table 2 also shows that the minority of the patients were smokers (4.9%), obese (16.1%), alcohol consumers (11.9%). More than 8 in 10 of the patients (81.8%) had never had any heart disease but almost 3 out of every 10 patients (27.3%) had diabetes Table 2. Bivariate analysis showed that the average LOS of males was higher by about 1.39 days than that of females while non-smokers (13.88 ± 9.01 days), diabetic patients (14.03 ± 9.11 days) and obese patients (13.83 ± 9.88 days) had higher LOS than those who were smokers, had no history of diabetes and not obese. However, none of these differences in means was found to be statistically significant. Similarly, currently unemployed patients had about 7 days more LOS on average than those that were currently employed. This difference in means was however, found to be statistically significant (t=3.08, 95% C. I= 2.38<*x*<10.94) Table 3. The difference in the average LOS of the different types of strokes was not found to be statistically significant (F=0.41, p= 0.663) with patients having ischemic stroke having the highest LOS (14.4± 9.8 days) and patients with hemispheric cerebrovascular stroke having the least average LOS (13.0 ± 7.7 days). Similarly, while increasing age groups had increasing average LOS with the exception of the oldest age group (70 years and older), the difference between these means was not statistically significant (F=1.88, p=0.136) Table 4.

**Table 1:** sociodemographic characteristics of patients

	n	%
**Sex**		
Male	76	53.1
Female	67	46.9
**Age group (years)**		
18-49	31	21.7
50-59	27	18.9
60-69	33	23.1
70 and older	52	36.4
**Religion**		
Christianity	97	67.8
Islam	46	32.2
**Marital Status**		
Currently Married	135	94.4
Currently Unmarried	8	5.6
**Occupation**		
Currently Unemployed	29	20.3
Currently Employed	114	79.7
**Types of Stroke**		
Ischemic Stroke	77	53.8
Hemorrhagic Stroke	26	18.2
Hemispheric Cerebrovascular disease	40	28.0
Mean age (± S.D)	61.5 ± 14.2 years	
Mean LOS (± S.D)	13.7± 8.9days	

**Table 2:** presence of risk factors and co-morbidities (N = 143)

	**n**	**%**
**Hypertension**		
Yes	109	76.2
No	34	23.8
**Diabetes**		
Yes	39	27.3
No	104	72.7
**Previous stroke incident**		
Yes	15	10.5
No	128	89.5
**Obesity**		
Yes	23	16.1
No	120	83.9
**Heart disease**		
Yes	26	18.2
No	117	81.8
**Smoking**		
Yes	7	4.9
No	136	95.1
**Alcohol Consumer**		
Yes	17	11.9
No	126	88.1

**Table 3:** association between socio-demographic characteristics and length of stay

	Mean ± S. D	t	95% Confidence Interval of the Difference	
Characteristics			Lower	Upper
**Sex**				
Male	14.79 ± 10.59	0.77	-2.17	4.94
Female	13.40 ± 10.87			
**Religion**				
Christianity	14.62 ± 11.56	0.78	-2.31	5.28
Islam	13.13 ± 8.67			
**Occupation**				
Currently unemployed	19.45 ± 16.69	3.08	2.38	10.94
Currently employed	12.79 ± 8.12			
**Alcohol consumption**				
Yes	13.41 ± 8.88	-0.15	-4.93	4.32
No	13.76 ± 8.98			
**Obese**				
Yes	13.83 ± 9.88	0.06	-3.91	4.16
No	13.70 ± 8.79			
**Smoking**				
Yes	10.71 ± 7.30	-0.91	-10.01	3.69
No	13.88 ± 9.01			
**Diabetes**				
Yes	14.03 ± 9.11	0.25	-2.91	3.75
No	13.61 ± 8.92			
**Previous heart disease**				
Yes	14.58 ± 9.83	0.59	-2.79	4.89
No	13.53 ± 9.55			

**Table 4:** one-way ANOVA of LOS between groups

Characteristics	Mean ± S. D	F	p-value
Types of Stroke			
Ischemic stroke	14.4 ± 9.8	0.41	0.663
Hemorrhagic stroke	13.0 ± 8.4		
Hemispheric Cerebrovascular disease	13.0 ± 7.7		
**Age Groups (years)**			
18-49	10.7 ± 5.7	1.88	0.136
50-59	14.0 ± 9.2		
60-69	15.9 ± 8.9		
70 and older	14.0 ± 8.4		

## Discussion

In this study, the majority of stroke patients were aged 60 and older. This is quite similar to other Nigerian studies. For example, Komolafe et al [16]. Reported that in South-West Nigeria, 76.1% of stroke patients were over 40 years old while in South-East Nigeria, about 62% of stroke patients were above 40 years. These findings agree with results from the world over which indicate that older people are at higher risk of suffering from stroke than younger people. However, it is important to note that the proportion of stroke patients under 40 years in this study is higher than the proportion of stroke patients in the same age bracket reported in the 1990s [17, 18]. This may be indicating that the epidemiology of stroke is gradually shifting from being a 'disease of the old' to that of the younger age group. The average LOS in this study was found to be far higher than that reported in other studies. For example, studied done in the United Kingdom and India reported an average LOS of less than 7 days in majority of the stroke patients [2, 19]. However, since these studies were carried out in countries believed to have better functioning health systems than Nigeria, these findings are understandable [17, 18]. Alkali et al in a 2013 study done in the country's capital city, Abuja (north central Nigeria)reported that the majority of stroke patients were admitted for more than 30 days suggesting that higher LOS reported in this study may be a common occurrence across the regions of the nation [14]. Out of 143 patient records assessed, 76 (53.1%) were males and 67 (46.9%) were females with a male to female ratio of 1: 0.9. This is similar to previous Nigerian studies that reported a male dominance [14, 20] but dissimilar to two studies, also from Nigeria, which reported a female dominance [21, 22]. The male sex has been listed in many studies as a risk factor for stroke. It was interesting to note that although not statistically significant, female stroke patients were slightly older than the males. This may be because females have a longer life expectancy than males [2, 19]. The presence of hypertension as the most common co-morbidity has also been found in other studies [16]. Hypertension, one of the most prevalent non-communicable conditions worldwide is responsible for an estimated 45% of deaths due to heart disease and 51% of deaths due to stroke. Hypertension is the main risk factor for stroke [23]. It has already been documented that hypertension is the dominant risk factor for stroke in Nigeria and its control has been associated with reduction in risk of stroke in other populations [24, 25]. However, just as Saxena et al also reported, the presence of hypertension was not statistically associated with LOS among stroke victims in this study [2].

Diabetes mellitus (DM) was the second prevalent risk factor among the patients in this study. This is similar to a study in the south west where DM was ranked second [22]. DM is known as a major risk factor for the development of atherosclerosis and the risk of stroke in diabetic patients is at least four times that of a normal individual in the general population [26]. Therefore, it is important to ensure good glycemic control in diabetic patients to prevent development of stroke. The average length of stay (ALOS) of stroke patients observed in this study is similar to previous studies in similar settings. For example, Desalu et al reported an average length of stay of 12 ± 10.7 days in a rural south-western hospital in Nigeria, which is just about 2 days less than what was reported in this study [22]. Alkali et al. also reported a mean hospital stay for stroke patients in National hospital in Abuja as 18.2 days with a median of 11 days [14]. When comparing this finding with what is obtainable among other LMICs, the LOS observed in Nigeria is still significantly higher than that found in Ghana where average LOS of 6.2 days have been reported but similar to Togo where average LOS of 17.4 days have been reported for stroke victims [27, 28]. Brainin has documented the variability in stroke case management peculiar to developing countries and which may result in wide variations in average LOS [29]. The strength of the study arises from its sample size which is higher than what have been reported by other studies in similar settings. This allows for greater variability in the sample. However, because the study was retrospective in nature and depended on hospital records, variables with incomplete data had to be removed from the final analysis. Problems with data collection in LMICs has been well documented in literature [30, 31].

## Conclusion

This study highlighted the susceptibility of a growing proportion of younger people to stroke. Thus, it is important to step up prevention measures such as promoting of healthier lifestyles to prevent a surge in the number of stroke cases. In addition, the average length of stay of stroke patients in Nigeria was shown to be prolonged especially when compared to similar settings in West Africa. The high prevalence of some of the risk factors of stroke such as diabetes mellitus indicates that policy and advocacy to drive changes in lifestyle are necessary to reduce the incidence of stroke and its consequent burden on health systems.

### What is known about this topic

- Stroke and infectious diseases are leading causes of death especially amongst the elderly;
- Severe systolic blood pressure, severe diastolic pressure, second or more episode of stroke, severe GCS, seizures, abnormal pupillary size, hemorrhagic stroke type, presence of aspiration pneumonitis, RBS > 200 mg/dl were independent predictors of mortality in stroke.

### What this study adds

- The average length of stay was 13.7 ± 8.9 days for stroke patients in Nigeria was shown to be prolonged especially when compared to similar settings in West Africa;
- Currently unemployed patients had about 7 days significantly more LOS on average than those that were currently employed;
- Though not significant, the difference in the average LOS showed patients having ischemic stroke as having the highest LOS (14.4± 9.8 days) and patients with hemispheric cerebrovascular stroke having the least average LOS (13.0 ± 7.7 days).
